# Supplementary material for: Continuous DNA Methylation Deconvolution-Based Surrogate for B-Cell Differentiation State in CLL
Source: bioRxiv. 2025 Sep 16:2025.09.16.676535. Preprint. [Version 1] doi: 10.1101/2025.09.16.676535 (PMC12458924; doi:10.1101/2025.09.16.676535)
Supplement: Supplement 1 [file media-1.pdf]

Supplementary Figures

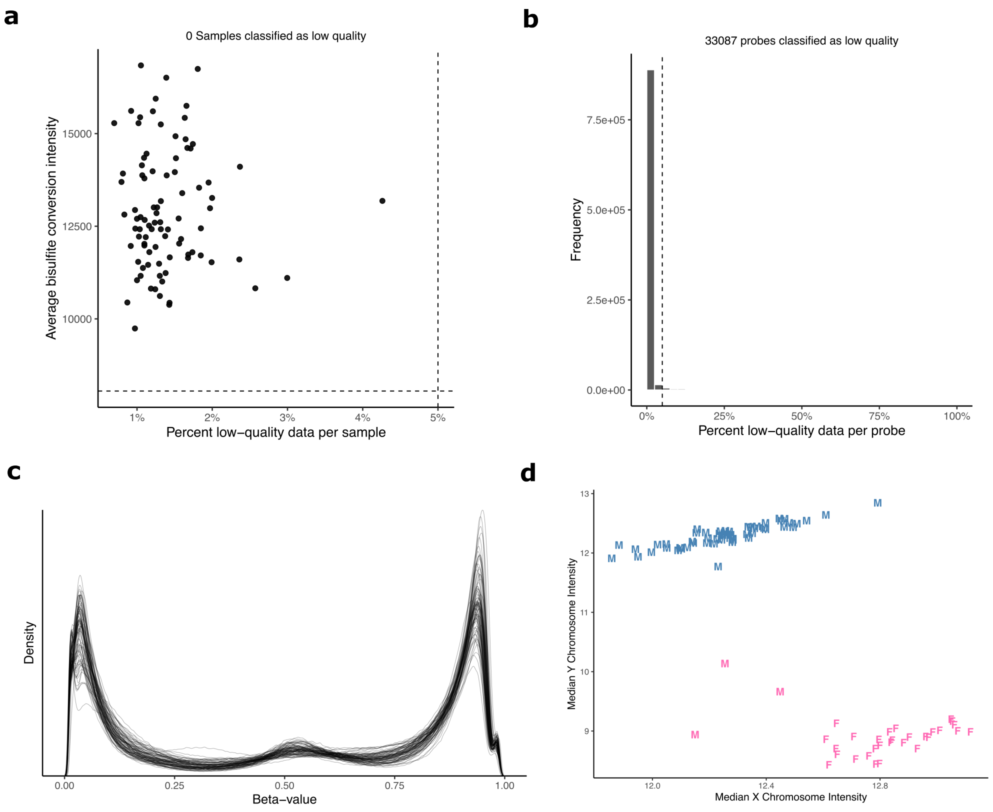

Supplementary Figure 1: Quality control on methylation data. a Quality control plot showing percentage of low-quality probes and average bisulfite conversion efficiency per sample. In a given sample, a low quality probe has either a detection p-value $> 10^{-6}$ or less than three beads measured. No samples exceed the exclusion threshold of >5% low quality probes, or exceeding three standard deviations from the population average bisulfite conversion intensity. **b** Per-probe plot, depicting proportions of samples where a given CpG is low quality. Probes deemed low quality in 5% or more of samples, as marked by the right side of the dashed line, were excluded. **c** Beta-value kernel density plot after quality control, filtering, and Normal-Out-Of-Band (NOOB) normalization ($n = 89$). **d** Plot showing sex misclassifications, through median X and Y chromosome intensity. Color corresponds to the methylation-based prediction and the letter (M or F) corresponds to the sample label. 3 samples labeled Male were predicted Female, and 0 labeled Female were predicted male. Maintaining a normal X chromosome intensity while losing Y chromosome intensity may be attributed to Y chromosome loss.

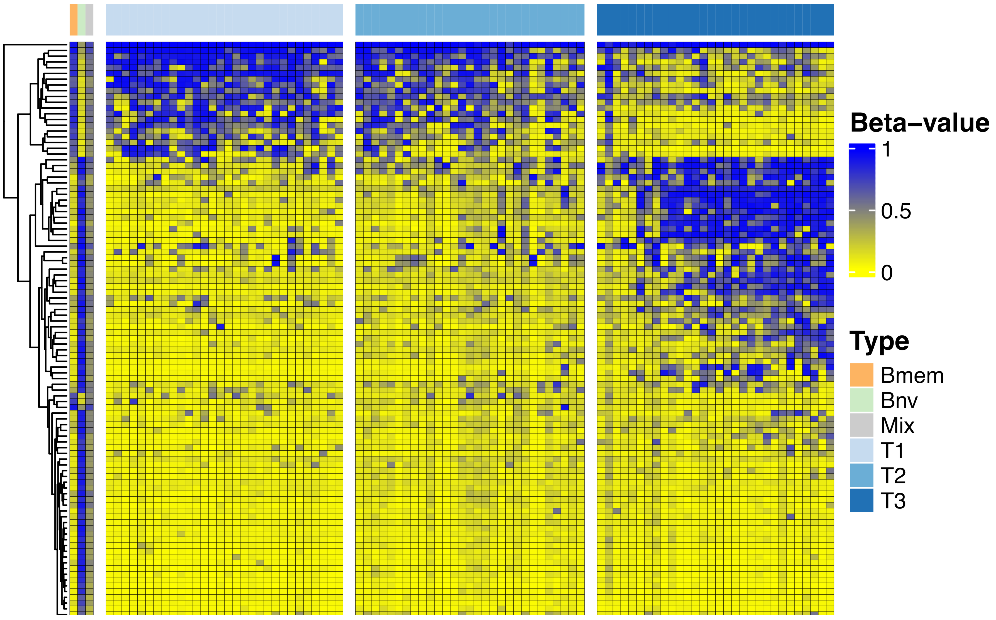

Supplementary Figure 2: Heatmap of CLL samples at B-memory and B-naive differentially methylated CpGs. Heatmap shows beta values of the 89 CLL samples, in order of decreasing B-memory proportion (the same order as Figure 1A), at the 100 most differentially methylated CpGs between B-naive and B-memory cells in the reference library. The right-most tertile is also shown in Figure 1C. As in Figure 1C, the pure B-memory and B-naive references are shown, as well as a 60:40 in-silico mixture of B-memory and B-naive references.

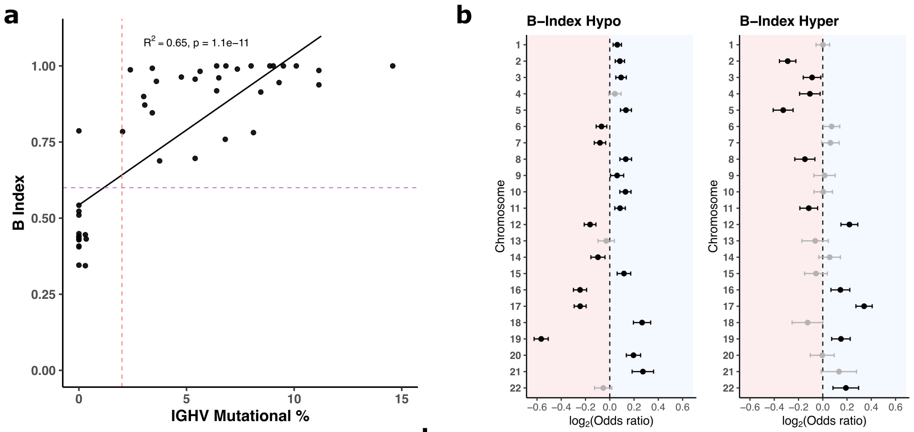

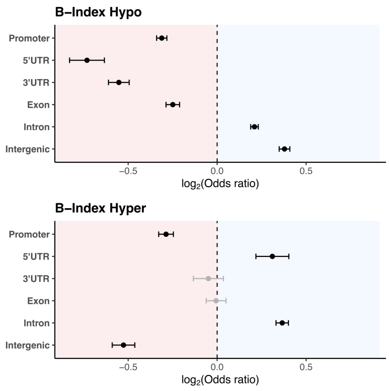

d

Methylation Direction	GO Term	P-value	FDR (BH)
Hypo	cell periphery	6.83e-48	1.52e-43
Hypo	plasma membrane	3.61e-39	4.02e-35
Hypo	signaling receptor activity	3.59e-20	2.00e-16
Hyper	regulation of developmental process	1.76e-07	3.91e-03
Hyper	plasma membrane	1.31e-06	1.42e-02
Hyper	cell periphery	2.47e-06	1.42e-02

e

Methylation Direction	KEGG Pathway	P-value	FDR (BH)
Hypo	Cornified envelope formation	4.68e-14	1.72e-11
Hypo	Cytokine-cytokine receptor interaction	6.08e-10	1.12e-07
Hypo	Cytoskeleton in muscle cells	1.20e-09	1.47e-07
Hyper	Phospholipase D signaling pathway	3.81e-04	1.39e-01
Hyper	Glycerolipid metabolism	7.59e-04	1.39e-01
Hyper	Pathways in cancer	1.64e-03	1.66e-01

Supplementary Figure 3: Validation and epigenetic correlates of B-Index. a Scatterplot of *IGHV* Mutational % and B-Index among 46 samples where percentage information was available. Horizontal purple dashed line is at optimal classification threshold for B-Index of 0.6. Vertical red dashed line is at 2%, the clinically used threshold for U-CLL and M-CLL classification. Black solid line is line of best fit. **b** Forest plot of chromosomal enrichment of hypo and hypermethylated CpGs from EWAS on B-Index. Odds ratio and 95% CI interval shown, in log_2 space. Entries in black are significant (FDR $p < 0.05$). **c** Forest plot of genomic context enrichment of hypo and hypermethylated CpGs from EWAS on B-Index, adjusting for CpG island relation using Cochran–Mantel–Haenszel (CMH) test. Odds ratio and 95% CI interval shown, in log_2 space. Entries in black are significant (FDR $p < 0.05$). **d** Top 3 hits from two GO pathway enrichment analysis conducted separately on the FDR significantly hypo and hypermethylated CpGs from EWAS on B-Index. **e** Top 3 hits from two KEGG pathway enrichment analysis conducted separately on the FDR significantly hypo and hypermethylated CpGs from EWAS on B-Index.

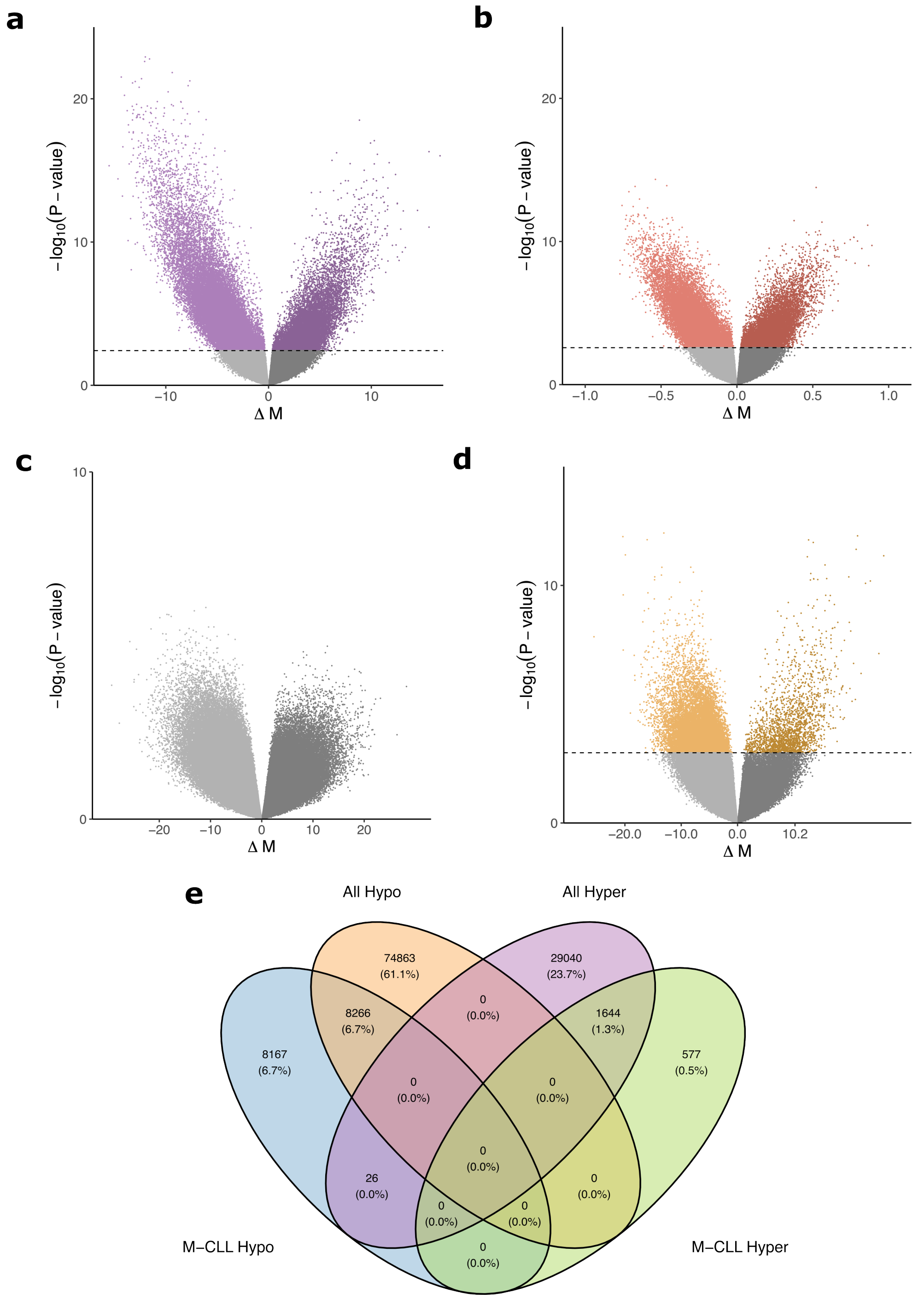

Supplementary Figure 4: B-Index compared to *IGHV* percentage and epigenetic signal of B-Index within subtypes. a EWAS of B-Index on cohort of 46 samples where *IGHV* percentage mutation was available, for comparison with (b). 14,776 CpGs were significantly hypermethylated (FDR p< 0.05), and 48,982 hypomethylated. **b** EWAS *IGHV* percentage mutation, for comparison with (a). 12,218 CpGs were significantly hypermethylated (FDR p< 0.05) and 32,250 hypomethylated. **c** EWAS on B-Index among the 24 U-CLL cases. No FDR-significant hits were observed. **d** EWAS on B-Index among the 56 M-CLL cases. 2,221 CpGs were FDR-significantly hypermethylated, and 16,459 were hypomethylated. **e** Venn diagram of CpGs identified by B-Index EWAS on full cohort, compared with B-Index EWAS within M-CLL subset.

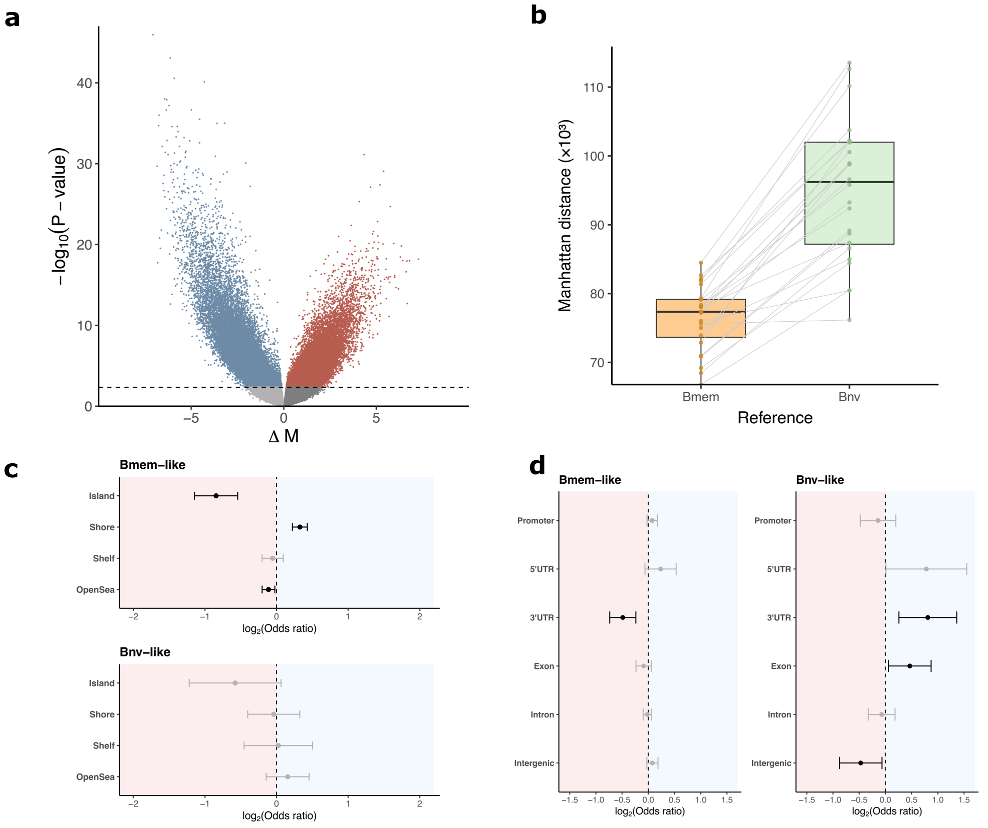

Supplementary Figure 5: Epigenetic similarities of U-CLL to B-memory and B-naive. a Volcano plot depicting EWAS between M-CLL and U-CLL. 28,462 were FDR-significantly hypermethylated, and 48,402 hypomethylated. **b** Box plots depicting the Manhattan distance of each U-CLL case to the mean B-memory reference, and mean B-naive reference at the full set of the 682,014 CpGs available across all data. Every U-CLL case has a higher distance from B-naive than B-memory. **c** Forest plot of CpG island context enrichment among CpGs in U-CLL similar (FDR $p < 0.05$) to B-memory and B-naive, adjusting for Illumina probe type using CMH Test. Odds ratio and 95% CI interval shown. Entries in black are significant (FDR $p < 0.05$). **d** Forest plot of genomic context enrichment among CpGs in U-CLL similar (FDR $p < 0.05$) to B-memory and B-naive, adjusting for CpG island relation using CMH test. Odds ratio and 95% CI interval shown. Entries in black are significant (FDR $p < 0.05$).

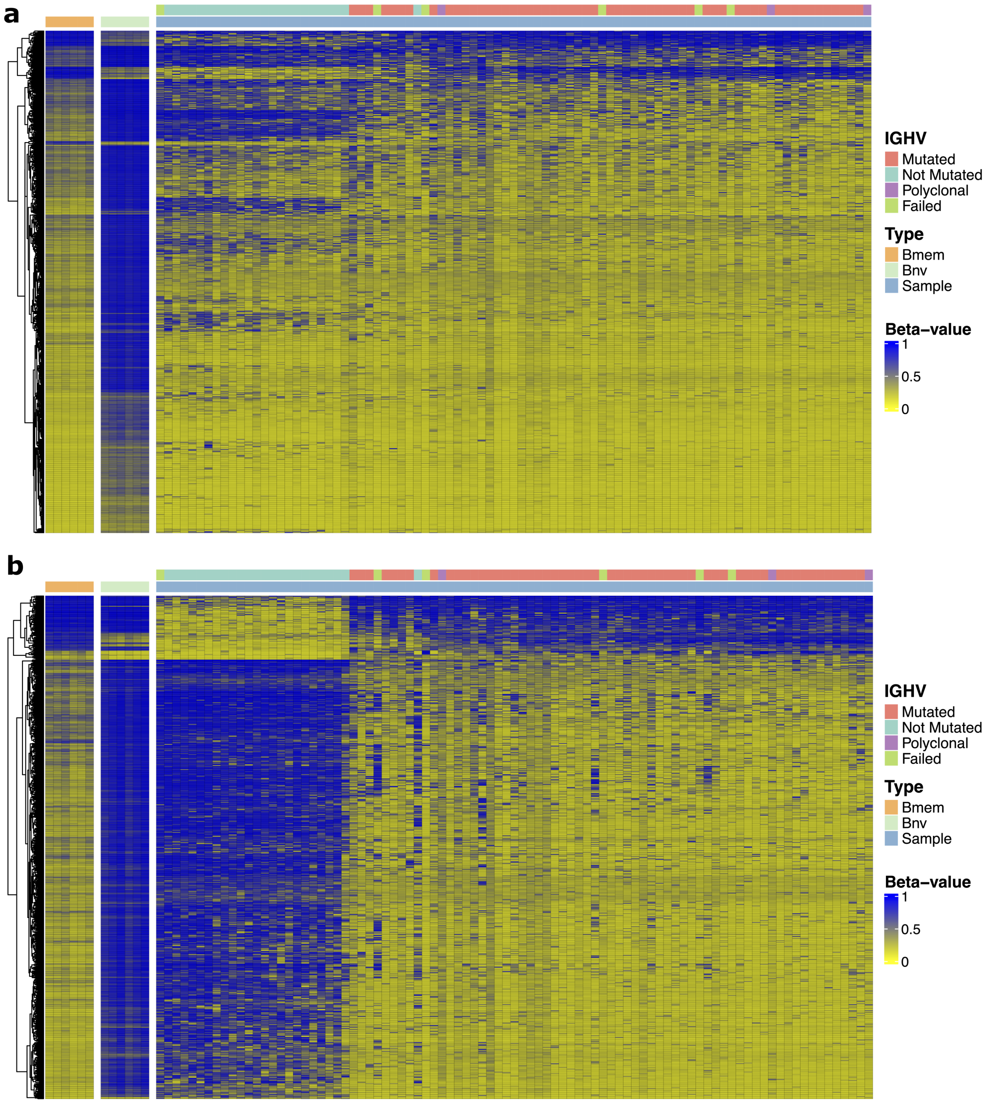

Supplementary Figure 6: Heatmaps of all CLL samples. **a** Heatmap of the top 1000 most significant CpGs from the B-memory v. B-naive EWAS available in our data. B-memory and B-naive references are shown alongside all samples, with IGHV mutational status shown in the top bar, and sorted in order of increasing B-Index from left to right. Heatmap contains the same CpGs from Figure 3B. **b** Heatmap similar to (a), except using top 1000 most CpGS from the U-CLL vs M-CLL EWAS. Heatmap contains the same CpGs from Figure 3C.

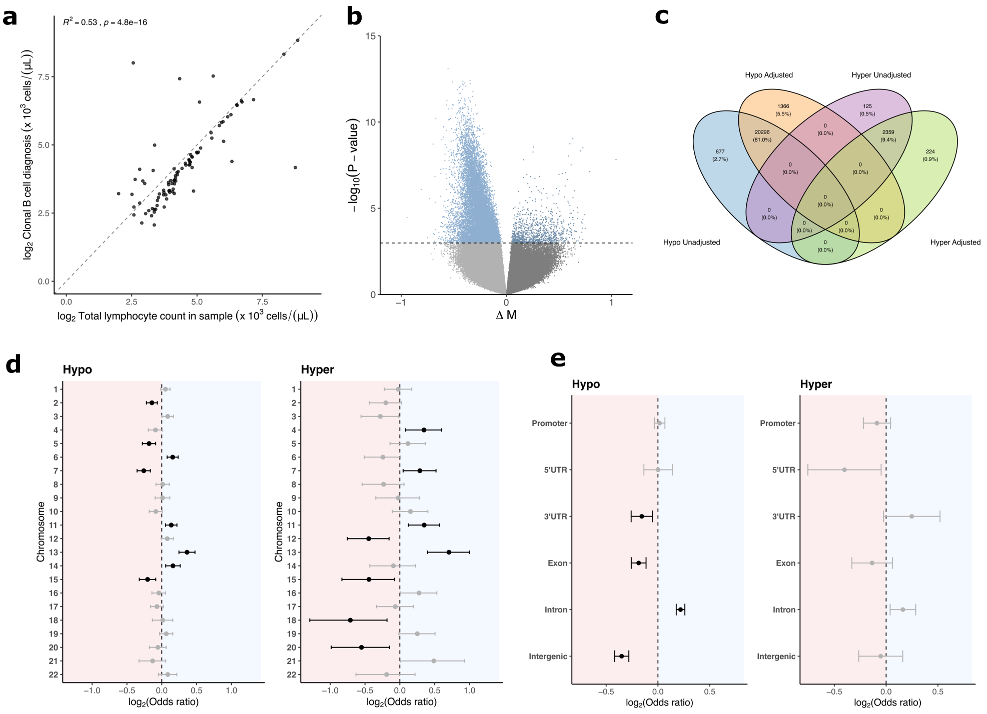

f

Methylation Direction	GO Term	P-value	FDR (BH)
Hypo	cytosol	1.83e-13	4.07e-09
Hypo	lymphocyte activation	4.67e-13	5.20e-09
Hypo	nucleoplasm	1.27e-12	9.41e-09
Hypo	positive regulation of immune response	6.40e-12	2.98e-08
Hyper	structural constituent of chromatin	8.12e-09	1.81e-04
Hyper	chromosome	3.69e-07	3.16e-03
Hyper	nucleosome	4.26e-07	3.16e-03
Hyper	meiotic chromosome segregation	3.11e-05	1.73e-01

Supplementary Figure 7: Epigenetic correlates of tumor burden. a Scatterplot between the two metrics for tumor burden: total lymphocyte count in sample, and clonal B-cell count at diagnosis (units of thousand cells per microliter). Correlation was conducted in log_2 space. R^2 was 0.53. **b** Volcano plot depicting EWAS of clonal B-cell count at diagnosis. **c** Venn diagram depicting the number of hypermethylated and hypomethylated FDR $p < 0.05$ significant CpGs from an EWAS on total lymphocyte count, compared to the same EWAS but adjusting for B-Index. **d** Forest plot of chromosomal enrichment among CpGs hypo and hypermethylated with increasing tumor burden. Odds ratio and 95% CI interval shown. Entries in black are significant (FDR $p < 0.05$). **e** Forest plot of genomic context enrichment among significantly (FDR $p < 0.05$) CpGs hypo and hypermethylated with increasing tumor burden, adjusting for CpG island relation using Cochran–Mantel–Haenszel (CMH) test. Odds ratio and 95% CI interval shown. Entries in black are significant (FDR $p < 0.05$). **f** Top 4 hits from gene ontology (GO) analysis on significantly (FDR $p < 0.05$) hypo and hypermethylated CpGs with increasing tumor burden.
